# History of a Case of Empyema from Protracted Measles and Pleurisy, in Which the Operation of Paracentesis Gave Immediate Relief

**Published:** 1829

**Authors:** Samuel Meriwether

**Affiliations:** Jeffersonville, Ind.


ART. VII.—*History of a case of Empyema from protracted Measles and Pleurisy, in which the operation of Paracentesis gave immediate relief.* By Dr. SAMUEL MERIWETHER, of Jeffersonville, Ind.

Calvin Cook, a youth of nineteen or twenty, of sanguineous temperament and delicate fibre, was attacked with measles some time last winter, perhaps in February, (1829) while at work on the Louisville and Portland Canal. He returned home to his mother's (Clark co. Ind.) where he remained a few weeks, when he was able to resume his labor. His cough remained, however, and from his exposed situation he was taken with Pleurisy, attended with violent pain in the left lobe of the lungs. From the best information I could gather, he was neglected or not regularly attended by his physician, and became emaciated, with cough, dyspnœa, a gradual enlargement of the left side, difficult respiration when placed on the right side, general debility, and hectic exascerbations.

On the third of May, I was called into consultation with Dr. Lewis. On examining the patient, I concurred with the doctor in the opinion, that an operation was the only means of relief that could be employed. The chylopoietic viscera appeared to be effected from contiguity or sympathy of parts, as there were considerable tension and tenderness at the pit of the stomach, with enlarged spleen; he was therefore directed to take a cathartic. On the morning of the 4th, Dr. Lewis visited the patient. The medicine had operated, and dejections from his bowels were healthy. During the evening he had two chills, with an entire absence of pain, &c. On the morning of the 5th, in company with Dr. Bridges, I visited our patient, and found his heart beating with considerable force on the right side, with general œdema of the left, which measured from the spinal column to the sternum nearly double that of the right, and had an indistinct fluctuation.

I gave the young man our opinion relative to his case, and that an operation was the only means of relief. To this he submitted with fortitude. Proceeding to the operation he was laid close to the edge of the bed, (previously made firm) with the affected side presenting. I made an incision between two and three inches long through the integuments, betwixt the sixth and seventh ribs, cutting close to the edge of the seventh, and carefully avoiding the intercostal artery; on the knife's passing through the intercostal muscles, the distension of the pleura costalis was very great, and on puncturing it a volume of pus issued forth, and continued to run for forty minutes; when his pulse became so languid as to require an immediate dressing of the wound. He expressed himself better under the discharge. We gave him an anodyne and directed wine and water to be given during the evening, should his pulse continue weak and low.

On the morning of the 6th, I found him every way better; he was free from fever, slept pretty well, had some appetite, and his respiration was easy, whether he lay on one side or the other. The distended ribs had subsided and likewise the œdema; the wound appearing to close, I introduced a blunt pointed probe and leaden canula about one and a half inches long, which kept up a discharge for two weeks at least.—The quantity of pus evacuated from first to last was judged, by all present, to be two gallons. From this time the patient's treatment was committed to the care of Dr. Lewis, by whom I have since been told, that the quantity of matter discharged while under his care, was equal to the first. His health is now fast improving, skin soft and perspirable, secretion from the kidneys and liver healthy, free from cough, wound entirely healed, and he takes gentle exercise every day on horseback or in an easy carriage. The general treatment has been aperients, elm or flax-seed tea, with Dover's powder occasionally at night, and lastly the sulphate of quinine as a tonic.

*June 10th*, 1829.

